# Preparing future dentists for artificial intelligence: a cross-sectional study of perceptions and educational needs in China

**DOI:** 10.3389/fpubh.2026.1841849

**Published:** 2026-05-19

**Authors:** Yanru Wu, Yusheng Bao, Ze Jiang, Hualing Sun

**Affiliations:** State Key Laboratory of Oral and Maxillofacial Reconstruction and Regeneration, Key Laboratory of Oral Biomedicine Ministry of Education, Hubei Key Laboratory of Stomatology, School and Hospital of Stomatology, Wuhan University, Wuhan, China

**Keywords:** artificial intelligence, China, dental education, dentistry, student attitudes

## Abstract

**Background:**

Artificial intelligence (AI) is entering dental education and clinical practice, yet little is known about how dental students at different stages of training understand its use, risks, and educational value. This study examined AI-related familiarity, attitudes, concerns, and learning needs among undergraduate, master’s, and doctoral dental students in China.

**Methods:**

A single-center cross-sectional online survey was conducted in July 2025 at the School and Hospital of Stomatology, Wuhan University. The 20-item questionnaire collected information on demographic characteristics, AI-related exposure, and perceived application areas, attitudes toward AI, main concerns, responsibility attribution, and educational needs. Descriptive statistics were used to summarize responses, and Pearson’s chi-square tests were used to compare differences across training stages.

**Results:**

A total of 343 valid responses were included, comprising 201 undergraduates, 92 master’s students, and 50 doctoral students. Few respondents described themselves as unfamiliar with AI, but high familiarity with dental AI remained uncommon. Overall, 89.8% supported the use of AI in dental care, and 84.2% expressed interest in AI education. Training-stage differences were found in information sources, perceived application domains, suitable stages for AI use in clinical care, responsibility attribution, and expectations for future AI development. Undergraduates more often learned about AI through formal courses and tended to place AI near learning support and diagnostic assistance. Master’s students showed greater acceptance of AI in treatment planning and implementation. Doctoral students were more cautious about AI in complex decision-making and gave more attention to responsibility, communication, and workload reduction. The most frequently reported concerns were data privacy and security, interpretability, and technological dependence.

**Conclusion:**

Dental students in this survey were generally open to AI, but their perceptions and educational needs differed across training stages. Dental AI education should move beyond general exposure and be aligned with students’ progression through training. Undergraduate teaching may focus on basic AI literacy and early critical awareness, master’s-level teaching on case-based evaluation within clinical workflows, and doctoral training on methodology, data governance, and ethical responsibility. Multicenter and longitudinal studies with practical assessments are needed to examine how AI-related competence develops during dental training.

## 1 Introduction

Artificial intelligence (AI) is no longer a distant topic in dentistry. Deep learning models have been applied to dental image interpretation, including caries detection and periapical lesion identification (1). Automated cephalometric analysis has also become part of digital orthodontic education and case planning (2). More recently, generative language models have widened students’ exposure to AI through literature searching, writing support, and learning tasks (3). Dental students may therefore encounter AI before they fully understand how such tools are built, validated, or limited. Knowing that an AI system can produce an answer is different from knowing whether that answer should be trusted. Curriculum proposals for dental AI have placed basic model concepts, validation, clinical interpretation, and responsible use at the center of AI education (4). Reviews of AI education in medical training, however, show that many programs still remain introductory and give less attention to clinical judgment, risk recognition, and interdisciplinary implementation (5, 6). Dental education now faces a practical question: how to prepare students to examine AI outputs rather than simply use them.

Students at different training stages encounter AI from different educational positions. Undergraduates are still building basic biomedical and dental knowledge, and AI may first appear to them as a tool for learning, searching, or diagnostic support. Master’s students have more contact with clinical rotations, specialty workflows, and research tasks, where AI may be judged by its usefulness in planning, documentation, and case discussion. Doctoral students are more likely to encounter questions of data quality, model reliability, research validity, clinical translation, and responsibility. Technology acceptance theories such as TAM and UTAUT are useful for understanding how perceived usefulness, prior exposure, and learning context may shape students’ willingness to use new tools (7, 8). Competency-based education adds another layer: professional ability develops through staged training, practical tasks, and observable performance (9). A single AI course for all students may therefore miss important differences in what learners need at each point in their education.

Existing surveys from South Korea, Iran, Egypt, and Palestine have shown that dental students often hold positive attitudes toward AI, while formal training, knowledge depth, and ethical awareness remain limited (10–13). These studies provide useful evidence, but many of them treat dental students as a broad group rather than examining how training stage changes students’ views of AI. Cross-country comparison also has limits. Dental curricula, clinical exposure, postgraduate pathways, and research requirements differ between education systems. In China, the five-year undergraduate program, master’s training, and doctoral training are separated by curriculum design, clinical involvement, and research expectations. Evidence from other systems cannot fully answer whether Chinese dental students at different stages differ in AI knowledge, application preferences, risk concerns, and educational needs.

To address this issue, we conducted a cross-sectional questionnaire survey among undergraduate, master’s, and doctoral students at the School and Hospital of Stomatology, Wuhan University. Training stage was used as the main comparison to examine students’ AI-related familiarity, information sources, perceived application areas, concerns, responsibility attribution, and learning needs. The study aimed to provide data for designing dental AI education that follows students’ training progression rather than treating all learners as a single group.

## 2 Methods

### 2.1 Study design and educational setting

A single-center, cross-sectional online survey was conducted in July 2025 at the School and Hospital of Stomatology, Wuhan University. The survey focused on undergraduate, master’s, and doctoral dental students and compared their AI-related knowledge, attitudes, concerns, and educational needs across training stages.

The School and Hospital of Stomatology, Wuhan University, provides undergraduate education, postgraduate training, clinical teaching, and research training. Students at different stages differ in curriculum exposure, clinical experience, research involvement, and professional responsibility. Training stage was therefore selected as the main grouping variable.

### 2.2 Participants, recruitment, and informed consent

Convenience sampling was used. Undergraduate, master’s, and doctoral students enrolled at the School and Hospital of Stomatology, Wuhan University during the survey period were invited to participate. The questionnaire link was distributed through teaching notification channels and online student communication platforms used within the school. One reminder was sent each day during the survey period.

The questionnaire was distributed through open institutional communication channels rather than an individually traceable student list. The exact response rate therefore could not be calculated. Before completing the questionnaire, participants read the study information and provided electronic informed consent. The survey was anonymous and voluntary. The study was approved by the Institutional Review Board of the School and Hospital of Stomatology, Wuhan University (Approval No. WDKQ2025-A23).

### 2.3 Questionnaire design and content

The questionnaire was developed from previous surveys on AI-related knowledge and attitudes in medical and dental education, with revisions for dental training in China. It was designed to assess not only general support for AI, but also students’ exposure to AI, perceived application areas, risk concerns, responsibility attribution, and learning needs.

The final questionnaire included 20 items: 4 demographic and educational items and 16 non-demographic items. Among the 16 non-demographic items, 8 were adapted from previous questionnaires, 4 were substantially modified from concepts used in earlier studies, and 4 were newly developed for the present study. The source and classification of each item are listed in Supplementary Table S1.

The questionnaire had three sections. The first section collected age, gender, training stage, and self-rated proficiency in using digital devices. The second section covered familiarity with AI, prior use of AI tools, information sources, perceived dental applications, and suitable clinical stages for AI use. The third section covered attitudes toward AI in dental care, major concerns, responsibility attribution, interest in AI learning, preferred timing of AI courses, future willingness to use or develop AI tools, and priority areas for improvement. The complete questionnaire is provided in Supplementary File S1.

### 2.4 Expert review, pilot testing, and questionnaire revision

The initial questionnaire was reviewed by three senior dental educators and two oral and maxillofacial surgeons. They evaluated the relevance of the items, clarity of wording, appropriateness of response options, and fit with dental education and clinical training in China.

A pilot test was conducted with 10 dental students. The pilot assessed item comprehensibility, completion time, and usability of the online survey platform. Based on expert and student feedback, several items and response options were revised before the final questionnaire was distributed.

### 2.5 Questionnaire distribution and data quality control

The questionnaire was distributed through Questionnaire Star (Changsha Ranxing Information Technology Co., Ltd., China). All items were set as mandatory to avoid missing responses. To reduce duplicate submissions, the platform restricted repeated responses from the same device and IP address.

No directly identifiable information, such as name, student ID number, or contact details, was collected. After data export, the dataset was checked for completeness. All valid questionnaires were included in the final analysis.

### 2.6 Reliability and assessment of questionnaire suitability

Questionnaire content was informed by previous literature, expert review, and pilot testing. Internal consistency was assessed using Cronbach’s alpha. The alpha value was 0.743, indicating acceptable internal consistency for an exploratory survey.

The Kaiser-Meyer-Olkin (KMO) test and Bartlett’s test of sphericity were used to assess whether the item set was suitable for examining item relationships. The KMO value was 0.823, and Bartlett’s test was significant (*p* < 0.001).

### 2.7 Statistical analysis

Data were analyzed using IBM SPSS Statistics 31.0. Categorical variables were summarized as frequencies and percentages. Age was reported as mean and standard deviation.

Pearson’s chi-square test was used to compare responses across training stages. For multiple-response questions, each option was coded as a separate binary variable. Likert-scale items were treated as ordinal categorical variables. All tests were two-sided, and *p* < 0.05 was considered statistically significant.

The analysis was designed to describe stage-related differences in AI-related knowledge, perceptions, and educational needs. No multivariable prediction model was fitted.

## 3 Results

### 3.1 Participant characteristics

A total of 343 valid questionnaires were analyzed. Participants included 201 undergraduates (58.6%), 92 master’s students (26.8%), and 50 doctoral students (14.6%). There were 140 males (40.8%) and 203 females (59.2%). The mean age was 22.82 years (SD = 3.29). Most participants rated themselves as “somewhat skilled” (45.8%) or “very skilled” (20.7%) in using personal computers or smartphones (Table 1).

**Table 1:** Responses to section 1 of the questionnaire: demographic characteristics (*N* = 343).

Characteristic	n or mean	% or SD
Age of students (Mean ± SD)	22.82	3.29
Gender		
Male	140	40.8
Female	203	59.2
Training stage		
Undergraduate	201	58.6
Master’s student	92	26.8
Doctoral student	50	14.6
Proficiency in using personal computers or smartphones		
Very unskilled	4	1.1
Somewhat unskilled	25	7.3
Average	86	25.1
Somewhat skilled	157	45.8
Very skilled	71	20.7

Data are presented as n (%) unless otherwise indicated. Age is presented as mean ± SD.

### 3.2 Awareness of AI

Most respondents did not describe themselves as unfamiliar with AI. Only 4.1% selected “unfamiliar” or “completely unfamiliar,” while 9.9% described themselves as “very familiar.” Familiarity with dental AI was lower; only 2.9% reported being “very familiar” with AI applications in dentistry (Table 2).

**Table 2:** Participants’ awareness of AI applications in dentistry (*N* = 343).

Question	Undergraduates	Master’s students	Doctoral students	Total	*p*
	*n* (%)				
1. How familiar are you with artificial intelligence (AI)?					
Very familiar	27(13.4)	3(3.3)	4(8.0)	34(9.9)	0.075
Fairly familiar	111(55.2)	55(59.7)	22(44.0)	188(54.8)	
Neutral	54(26.9)	30(32.6)	23(46.0)	107(31.2)	
Unfamiliar	8(4.0)	3(3.3)	1(2.0)	12(3.5)	
Completely unfamiliar	1(0.5)	1(1.1)	0(0.0)	2(0.6)	
2. How familiar are you with AI applications in dentistry?					
Very familiar	5(2.5)	2(2.2)	3(6.0)	10(2.9)	0.859
Fairly familiar	74(36.8)	36(39.1)	18(36.0)	128(37.3)	
Neutral	99(49.3)	48(52.2)	24(48.0)	171(49.9)	
Unfamiliar	17(8.4)	5(5.4)	4(8.0)	26(7.6)	
Completely unfamiliar	6(3.0)	1(1.1)	1(2.0)	8(2.3)	
3. Which developmental stage do you think AI currently occupies in dentistry?					
Conceptual stage, with minimal applications	9(4.5)	1(1.1)	3(6.0)	13(3.8)	0.260
Preliminary application stage, mainly in diagnostic assistance or research	103(51.2)	54(58.7)	29(58.0)	186(54.2)	
Rapid development stage, with increasingly widespread applications	81(40.3)	35(38.0)	16(32.0)	132(38.5)	
Mature application stage, deeply integrated into clinical workflows	1(0.5)	2(2.2)	0(0.0)	3(0.9)	
Unclear	7(3.5)	0(0.0)	2(4.0)	9(2.6)	
4. Have you ever used any artificial intelligence software, apps, or mini programs for dentistry?					
Proficient in commonly used artificial intelligence software	9(4.5)	8(8.7)	7(14.0)	24(7.0)	0.136
Able to use some software features independently	65(32.3)	37(40.2)	16(32.0)	118(34.4)	
Unable to use the software independently; guidance is required	56(27.9)	20(21.7)	9(18.0)	85(24.8)	
Never used	71(35.3)	27(29.4)	18(36.0)	116(33.8)	
5. How did you learn about the application of artificial intelligence in dentistry?					
Professional academic journals or conferences	76(37.8)	56(60.9)	26(52.0)	158(46.1)	<0.001^**^
Industry news or professional media	60(29.9)	41(44.6)	20(40.0)	121(35.3)	0.038^*^
Continuing education training or lectures	30(14.9)	18(19.6)	6(12.0)	54(15.7)	0.440
Medical school curriculum	133(66.2)	38(41.3)	13(26.0)	184(53.6)	<0.001^**^
Peer exchange	79(39.3)	45(48.9)	21(42.0)	145(42.3)	0.303
Promotion by related product or software companies	35(17.4)	27(29.3)	10(20.0)	72(21.0)	0.065
Social media or web searches	119(59.2)	48(52.2)	22(44.0)	189(55.1)	0.124
Little or no understanding	20(10.0)	2(2.2)	4(8.0)	26(7.6)	0.065
6. Based on your understanding, in which specific areas of dentistry can AI be applied?					
Oral imaging analysis	164(81.6)	80(87.0)	40(80.0)	284(82.8)	0.450
Caries risk assessment and radiographic diagnosis	125(62.2)	62(67.4)	24(48.0)	211(61.5)	0.073
Periodontal disease radiographic diagnosis and status assessment	105(52.2)	55(59.8)	19(38.0)	179(52.2)	0.046^*^
Early screening for oral cancer	113(56.2)	51(55.4)	20(40.0)	184(53.6)	0.111
Orthodontic treatment plan design and simulation	133(66.2)	68(73.9)	22(44.0)	223(65.0)	<0.001^**^
Implant surgery planning and surgical guide design	119(59.2)	65(70.7)	29(58.0)	213(62.1)	0.140
Computer-aided design and manufacturing (CAD/CAM) of prostheses	132(65.7)	70(76.1)	28(56.0)	230(67.1)	0.042^*^
Radiological examination of jawbone lesions	71(35.3)	41(44.6)	10(20.0)	122(35.6)	0.014^*^
Intelligent management of patient medical records	110(54.7)	58(63.0)	19(38.0)	187(54.5)	0.017^*^
Virtual assistant/chatbot	117(58.2)	58(63.0)	22(44.0)	197(57.4)	0.085
Clinical decision support system	87(43.3)	41(44.6)	14(28.0)	142(41.4)	0.112
Teaching and training simulation	120(59.7)	47(51.1)	26(52.0)	193(56.3)	0.311
I am not sure	7(3.5)	4(4.3)	1(2.0)	12(3.5)	0.767
7. In your opinion, which of the following stages of oral treatment are most suitable for the introduction of AI?					
Diagnostic phase	150(74.6)	67(72.8)	39(78.0)	256(74.6)	0.795
Treatment planning phase	134(66.7)	70(76.1)	21(42.0)	225(65.6)	<0.001^**^
Treatment implementation phase	101(50.2)	64(69.6)	22(44.0)	187(54.5)	0.002^**^
Post-treatment outcome evaluation and follow-up tracking	126(62.7)	54(58.7)	21(42.0)	201(58.6)	0.029^*^
Patient communication and education	103(51.2)	59(64.1)	33(66.0)	195(56.9)	0.044^*^
Clinic management process	137(68.2)	59(64.1)	28(56.0)	224(65.3)	0.261
I am not sure	4(2.0)	1(1.1)	1(2.0)	6(1.7)	0.852

*p < 0.05, **p < 0.01.

More than half of the respondents (54.2%) considered dental AI to be at a preliminary application stage, mainly in diagnostic assistance or research. Another 38.5% viewed it as being in a rapid development stage. Regarding prior use of AI-related tools, 33.8% had never used such tools, while 7.0% reported proficient use.

### 3.3 Information acquisition channels

Sources of AI-related information differed across training stages (Table 2). Undergraduates most often reported medical school curriculum (66.2%) and social media or web searches (59.2%) as their information sources. Master’s and doctoral students more often selected professional academic journals or conferences (60.9 and 52.0%, respectively; *p* < 0.001). They also reported greater use of industry news or professional media (44.6 and 40.0%, respectively; *p* = 0.038).

### 3.4 Recognition of AI application domains

The most frequently recognized application areas were oral imaging analysis (82.8%), CAD/CAM of prostheses (67.1%), orthodontic treatment planning and simulation (65.0%), and implant surgery planning and surgical guide design (62.1%).

Responses differed by training stage for several application domains, including periodontal disease radiographic diagnosis and status assessment (*p* = 0.046), orthodontic treatment planning and simulation (*p* < 0.001), CAD/CAM of prostheses (*p* = 0.042), radiological examination of jawbone lesions (*p* = 0.014), and intelligent management of patient medical records (*p* = 0.017).

### 3.5 Perceived role of AI in diagnostic and treatment processes

The diagnostic phase was the most frequently selected stage for AI integration (74.6%), followed by treatment planning (65.6%) (Table 2). Responses differed across training stages for treatment planning (*p* < 0.001), treatment implementation (*p* = 0.002), post-treatment evaluation and follow-up tracking (*p* = 0.029), and patient communication and education (*p* = 0.044).

### 3.6 Recognition of AI’s potential value

**Figure 1:**
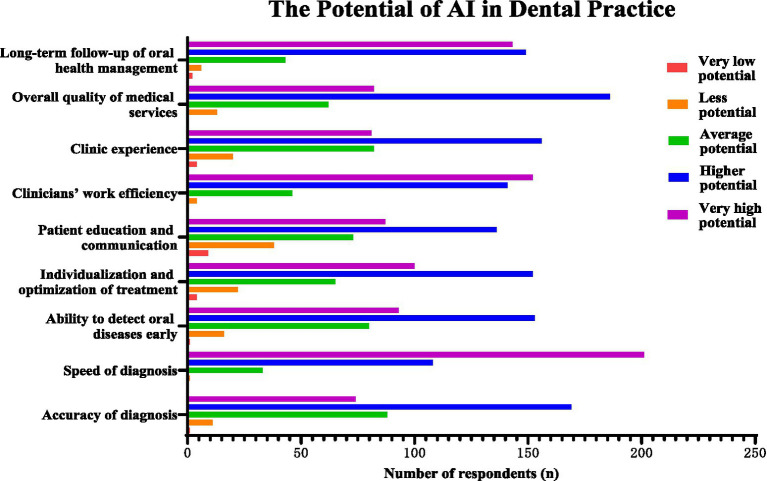
Number of respondents selecting each response category for the perceived potential of AI in different aspects of dental care. Responses were rated on a five-point scale from “very low potential” to “very high potential.” Data are presented as the number of respondents (*n*).

Figure 1 shows respondents’ ratings of the potential of AI to improve different aspects of dental care. The most frequently selected perceived benefit of AI was faster diagnosis (58.6%). Other common responses were improving clinicians’ work efficiency (44.3%) and supporting long-term oral health management (41.7%). A smaller proportion of participants (13.7%) considered AI to have limited potential in patient education and communication.

### 3.7 Attitudes toward AI applications

Overall support for AI in dentistry was high. In total, 89.8% of students selected “very supportive” or “relatively supportive.” Neutral or concerned responses accounted for 9.9% (Table 3).

**Table 3:** Participants’ attitudes toward and acceptance of AI applications in dentistry (*N* = 343).

Question	Undergraduates	Master’s students	Doctoral students	Total	*p*
	*n* (%)				
1. In general, what is your stance on applying AI technology to the field of dental care?					
Very supportive	79(39.3)	38(41.3)	18(36.0)	135(39.4)	0.947
Relatively supportive	104(51.7)	44(47.8)	25(50.0)	173(50.4)	
Neutral or indifferent	14(7.0)	9(9.8)	6(12.0)	29(8.4)	
A bit worried	3(1.5)	1(1.1)	1(2.0)	5(1.5)	
Inconclusive	1(0.5)	0(0.0)	0(0.0)	1(0.3)	
2. When AI makes a mistake in the course of treatment, who should be responsible?					
AI developers	19(9.5)	7(7.6)	13(26.0)	39(11.4)	0.005^**^
Doctors	5(2.5)	4(4.3)	2(4.0)	11(3.2)	
Medical organizations	32(15.9)	7(7.6)	3(6.0)	42(12.2)	
Developers, doctors, and medical organizations should share responsibility	145(72.1)	74(80.4)	32(64.0)	251(73.2)	
3. Are you personally interested in learning or acquiring knowledge and skills related to the application of AI in the field of dentistry?					
Very interested	55(27.4)	25(27.2)	10(20.0)	90(26.2)	0.138
Somewhat interested	121(60.2)	52(56.5)	26(52.0)	199(58.0)	
Generally interested	23(11.4)	12(13.0)	13(26.0)	48(14.0)	
Not really interested	2(1.0)	1(1.1)	0(0.0)	3(0.9)	
No interest at all	0(0.0)	0(0.0)	0(0.0)	0(0.0)	
Inconclusive	0(0.0)	2(2.2)	1(2.0)	3(0.9)	
4. At which stage of study do you think a course on AI applications would be most helpful?					
It should be included in undergraduate dental education	77(38.3)	31(33.7)	19(38.0)	127(37.0)	0.492
It should be included in master’s-level dental education	67(33.3)	31(33.7)	10(20.0)	108(31.5)	
It should be included in doctoral dental education	9(4.5)	7(7.6)	2(4.0)	18(5.2)	
It should be included in professional training for dentists	39(19.4)	19(20.7)	15(30.0)	73(21.3)	
Currently unknown	9(4.5)	4(4.3)	4(8.0)	17(5.0)	
5. Are you willing to try applying AI tools or collaborating in the development of AI tools in future clinical practice or research?					
Very willing and looking forward to it	68(33.8)	31(33.7)	17(34.0)	116(33.8)	0.176
More willing to try	87(43.3)	43(46.7)	18(36.0)	148(43.1)	
Neutral, depending on the specific situation	45(22.4)	17(18.5)	12(24.0)	74(21.6)	
Not really willing to try	1(0.5)	0(0.0)	2(4.0)	3(0.9)	
Very reluctant	0(0.0)	0(0.0)	0(0.0)	0(0.0)	
Inconclusive	0(0.0)	1(1.1)	1(2.0)	2(0.6)	
6. When do you think AI will have a significant impact on the field of dentistry?					
Within 1 year	4(2.0)	4(4.3)	2(4.0)	10(2.9)	0.769
Within 1–5 years	108(53.7)	50(54.3)	24(48.0)	182(53.1)	
Within 5–10 years	75(37.3)	28(30.4)	19(38.0)	122(35.6)	
More than 10 years	9(4.5)	8(8.7)	4(8.0)	21(6.1)	
Currently unknown	5(2.5)	2(2.2)	1(2.0)	8(2.3)	
7. What is the most important aspect of dentistry that you would like to see AI address or improve?					
Improving diagnosis of certain diseases	62(30.9)	13(14.1)	12(24.0)	87(25.4)	0.011^*^
Optimizing complex surgical steps	32(15.9)	23(25.0)	3(6.0)	58(16.9)	
Reducing the paperwork burden	72(35.8)	28(30.4)	25(50.0)	125(36.4)	
Improving patient management efficiency	30(14.9)	24(26.1)	8(16.0)	62(18.1)	
Providing supplementary instruction	3(1.5)	2(2.2)	1(2.0)	6(1.7)	
Other	2(1.0)	2(2.2)	1(2.0)	5(1.5)	

*p < 0.05, **p < 0.01.

### 3.8 Concerns regarding AI applications

**Figure 2:**
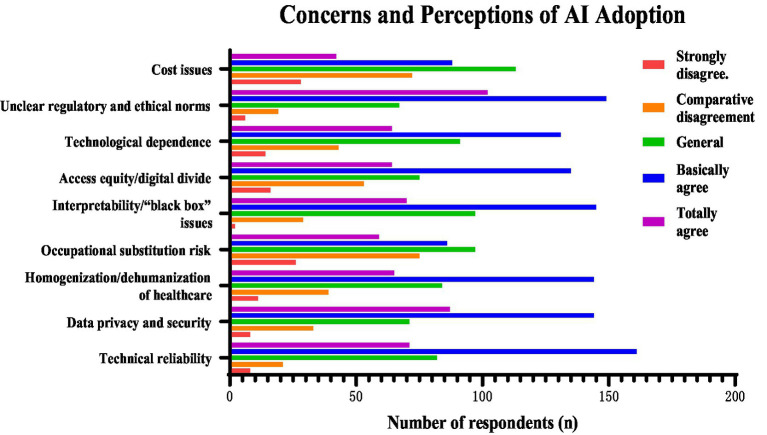
Number of respondents selecting each response category for concerns about AI applications in dentistry. Responses were rated on a five-point agreement scale from “strongly disagree” to “totally agree.” Data are presented as the number of respondents (*n*).

The most frequently reported concerns were data privacy and security, interpretability, and technological dependence (Figure 2). The proportions of respondents rating these concerns as 4 or 5 on the five-point agreement scale were 67.3, 62.7, and 56.9%, respectively. No significant differences were found across training stages.

### 3.9 Responsibility attribution

When asked about responsibility for AI-related errors, 73.2% of participants selected shared responsibility among AI developers, doctors, and medical organizations. Responsibility attribution differed across training stages (*p* = 0.005). Doctoral students more often selected AI developers as solely responsible (26.0%), while undergraduates more often selected medical organizations (15.9%).

### 3.10 Willingness to learn and apply AI

Most participants were interested in AI education. In total, 84.2% selected “very interested” or “somewhat interested.” Willingness to try AI tools or collaborate in AI tool development was also high, with 77.0% selecting “very willing and looking forward to it” or “more willing to try.”

Regarding the timing of AI courses, 37.0% preferred inclusion during undergraduate education, and 31.5% preferred inclusion during master’s-level education.

### 3.11 Expectations for AI development

More than half of the respondents (53.1%) expected AI to have a significant impact on dentistry within 1–5 years, and 35.6% expected this impact within 5–10 years.

When asked which area of dentistry they most wanted AI to improve, the most common response was reducing paperwork burden (36.4%), followed by improving diagnosis of certain diseases (25.4%). Responses differed across training stages (*p* = 0.011). Doctoral students more often emphasized reducing paperwork burden (50.0%), while master’s students more often selected optimization of complex surgical steps (25.0%).

## 4 Discussion

This survey points to a gap between openness to AI and the kind of judgment that dental education ultimately needs to build. Students rarely rejected AI, but few reported high familiarity with dental applications. The useful message is therefore not simply that dental students are interested in AI. Rather, dental education needs to consider whether students can examine AI-assisted outputs, understand their limits, and place them within clinical and professional responsibility.

The stage-related pattern was not a simple ladder from lower to higher acceptance. Undergraduates tended to place AI near learning support and diagnostic assistance. Master’s students were more receptive to AI in treatment planning and implementation. Doctoral students were more selective about AI in complex clinical tasks and gave more attention to communication, workload reduction, and responsibility. These differences provide the basis for discussing staged AI education, but they should not be read as fixed learner categories.

### 4.1 Positive attitudes do not necessarily indicate AI competence

Most respondents supported the use of AI in dentistry and expressed interest in learning AI-related knowledge and skills. Their familiarity with dental AI was much lower. Only a small proportion described themselves as very familiar with AI applications in dentistry. A positive attitude toward AI is therefore better understood as an educational starting point, not as evidence of clinical readiness.

Dental AI is already visible in imaging analysis, orthodontic assessment, implant planning, documentation, and patient communication. Recognition, however, is a shallow endpoint. Students need to understand how training data, validation methods, target populations, and clinical context affect AI outputs before they can decide whether an AI result is useful for a particular patient. Curriculum recommendations for AI in oral and dental healthcare also place critical appraisal, responsible use, and clinical interpretation alongside basic knowledge of AI applications (4).

Similar problems have been reported in health-profession education. A multinational survey found that many medical and dental students supported AI education, while formal training remained limited (14). A systematic review and meta-analysis also reported positive attitudes toward AI among medical, dental, and nursing students, together with insufficient knowledge in ethics and human–AI collaboration (15). In the present survey, willingness to learn AI was already high, but the ability to judge AI-assisted decisions cannot be assumed from willingness alone.

AI teaching in dentistry needs to move beyond exposure. Courses should not only introduce common applications or encourage interest in new tools. They should train students to compare AI outputs with clinical evidence, teacher feedback, and patient-specific conditions. In this sense, AI should be learned as a tool to be questioned and tested, not as a result to be accepted.

### 4.2 Stage-related differences in how students frame AI

Training stage was associated with different ways of placing AI within dental learning and practice. The differences were not simply differences in enthusiasm. Undergraduates appeared to place AI close to learning and early diagnostic support, whereas master’s students viewed it more through clinical workflow. Doctoral students, in contrast, seemed less concerned with whether AI was useful in general than with where its use might become unsafe or professionally ambiguous. These differences likely reflect educational exposure rather than fixed learner types.

Among undergraduates, the information pathway was still close to the curriculum and to general online sources. Their responses focused on familiar applications such as imaging analysis, diagnostic support, and early learning assistance. At this stage, students are still building basic dental knowledge and have limited responsibility for independent clinical judgment. AI is more likely to be understood as a tool for information access, recognition, and preliminary learning.

For master’s students, the pattern was closer to clinical workflow. They relied more on academic journals, conferences, and professional media, and they showed greater acceptance of AI in treatment planning and treatment implementation. Their contact with specialty training and clinical case discussion may make the practical value of AI more visible. For this group, AI is more likely to be judged through planning, documentation, communication, and workflow support.

Doctoral students were more restrained in judging where AI should be used. They were less accepting of AI involvement in treatment planning, treatment implementation, and outcome evaluation, but placed greater emphasis on patient communication and reducing documentation burden. The doctoral response does not indicate rejection of AI. It points to caution when AI is placed near complex clinical judgment, and to greater interest in supportive roles that reduce routine workload without replacing professional decision-making.

### 4.3 Responsibility attribution and professional judgment in AI-assisted care

Most respondents selected shared responsibility among AI developers, doctors, and medical organizations when asked about AI-related errors. This answer is reasonable, but it needs to be made operational in education. AI-assisted care combines technical design, clinical interpretation, and institutional oversight. Developers are responsible for data quality, model training, validation, and technical safety. Clinicians remain responsible for patient assessment, clinical judgment, and communication. Medical organizations decide how AI tools are introduced, supervised, and restricted in practice. Without these distinctions, shared responsibility can remain too broad to guide real clinical decisions.

The stage-related difference in responsibility attribution was mainly reflected in doctoral students’ stronger tendency to select AI developers. This may be related to their greater exposure to research design, data analysis, and model evaluation. Students who have encountered research workflows may be more sensitive to problems arising before clinical use, such as biased datasets, limited external validation, poor interpretability, or weak reporting. Ethical discussions of machine learning in healthcare have also emphasized bias, transparency, responsibility allocation, and the clinician–patient relationship as central issues in clinical AI implementation (16).

Responsibility education in dental education needs to be linked to concrete tasks. Undergraduate students need to understand privacy, informed use, and the clinician’s duty to verify AI outputs. Master’s students need case-based discussion of conflicts between AI recommendations and clinical judgment. Doctoral students need deeper training in data governance, model validation, reporting standards, and responsibility in AI research translation. Responsibility education should be part of clinical and research training, rather than a separate list of general warnings.

### 4.4 A preliminary staged approach to AI education in dentistry

Table 4 presents a preliminary framework for AI education in dentistry. It is a teaching proposal, not a validated curriculum model. Its purpose is to help dental schools move from general AI awareness to stage-appropriate training. The framework is meant to match AI teaching with students’ actual learning tasks, rather than adding a single general AI course for all students.

**Table 4:** A preliminary staged framework for AI education in dental training.

Training stage	Suggested educational focus	Suggested learning objectives and teaching approaches
Undergraduate students	Basic AI literacy and early critical awareness	Recognize common dental AI applications; understand basic concepts of data sources, model outputs, and clinical limitations; develop early awareness of privacy, ethical use, and the need to verify AI-generated information. AI appraisal tasks can be embedded into dental radiology, orthodontics, prosthodontics, and introductory clinical courses.
Master’s students	Case-based clinical evaluation and workflow integration	Compare AI-generated outputs with radiographs, patient records, teacher feedback, and treatment plans; identify discrepancies between AI outputs and clinical judgment; judge appropriate use boundaries in diagnosis, planning, documentation, and communication. Case conferences, specialty seminars, and clinical rotations can be used for structured evaluation of AI outputs.
Doctoral students	AI methodology, data governance, and research translation	Critically appraise AI-related studies; understand model training, validation, bias, external applicability, and reporting standards; consider data governance, responsibility allocation, and the translation of AI tools into clinical or research settings. Journal clubs and research-methods courses can include appraisal of AI study design, validation, reporting quality, and ethical responsibility.

For undergraduates, AI education can begin with basic literacy and early critical awareness. These topics do not need to be taught only as a separate technical module. Dental radiology can introduce AI-assisted image interpretation; orthodontics and prosthodontics can discuss automated assessment or design tools; and introductory clinical courses can ask students to compare AI-generated information with textbook knowledge and teacher feedback. At this stage, the main aim is not to train students to develop AI models, but to help them recognize common applications and understand why AI outputs require verification.

For master’s students, AI teaching should move closer to clinical cases and specialty workflows. Case conferences, specialty seminars, and clinical rotations can include structured comparison between AI-generated outputs, radiographs, patient records, teacher feedback, and treatment plans. The emphasis should be on judging when AI is useful, when its output is uncertain, and when clinical judgment should take priority.

For doctoral students, the curriculum can place greater weight on methodology, data governance, and clinical translation. Journal clubs and research-methods courses can include model validation, bias, external applicability, reporting standards, and responsibility in AI-related research. This training is especially relevant for students who may later participate in AI development, evaluation, or implementation.

The framework requires local adaptation and educational evaluation. Dental schools differ in curriculum structure, clinical exposure, digital infrastructure, and faculty expertise. A practical first step would be to embed short AI appraisal tasks into existing courses before building larger standalone modules. Later studies can examine whether staged AI training improves students’ ability to evaluate AI outputs, handle ethical issues, and use AI safely in clinical learning. Existing curriculum recommendations for AI in oral and dental healthcare provide a useful reference for this work (4).

### 4.5 Strengths and limitations

This study compared undergraduate, master’s, and doctoral dental students within the same institution. The shared educational setting allowed a focused comparison across training stages. The questionnaire also covered several dimensions of AI-related education, including familiarity, information sources, perceived applications, responsibility attribution, and learning needs, rather than measuring attitude alone.

The study was conducted at a single dental school and used convenience sampling, which limits generalizability. Students from institutions with different curricula, digital infrastructure, clinical exposure, or AI teaching resources may respond differently. The questionnaire was distributed through open institutional communication channels, and the exact response rate could not be calculated. Students with stronger interest in AI or digital technologies may have been more likely to participate. The data were self-reported, so the results describe perceptions and willingness rather than actual ability to evaluate or use AI tools.

The statistical analysis was planned to describe stage-related differences rather than build prediction models. Training stage may be linked with digital proficiency, previous AI use, research exposure, and clinical experience, so the observed associations should be interpreted as exploratory. Future studies should also report effect size estimates to better contextualize statistically significant differences. The cross-sectional design also prevents conclusions about how AI literacy develops during dental training. Longitudinal studies with case-based or practical assessments are needed to examine whether staged AI education improves students’ ability to evaluate AI outputs and use AI responsibly.

## 5 Conclusion

This survey does not show that dental students are ready for AI-assisted care simply because they are interested in AI. It shows that they are receptive to AI, but differently positioned to learn it. Undergraduates tended to place AI near learning support and diagnostic assistance. Master’s students were more receptive to AI in treatment planning and implementation. Doctoral students were more cautious about AI in complex decision-making and paid closer attention to communication, workload reduction, and responsibility.

Dental AI education should not stop at awareness-raising. Undergraduate teaching can build basic AI literacy and early critical awareness within existing courses. Master’s-level training can use clinical cases to evaluate AI outputs within real workflows. Doctoral education can address AI methodology, data governance, clinical translation, and ethical responsibility.

The results are exploratory because the study was single-center, cross-sectional, and based on self-reported data. Future multicenter and longitudinal studies with practical assessments are needed to examine how AI-related competence develops during dental training and how staged educational interventions affect students’ ability to use AI safely and critically.

## Data Availability

The original contributions presented in the study are included in the article/Supplementary material, further inquiries can be directed to the corresponding author.
