# Supplementary material for: Preparing future dentists for artificial intelligence: a cross-sectional study of perceptions and educational needs in China
Source: Front Public Health. 2026 May 19;14:1841849. doi: 10.3389/fpubh.2026.1841849 (PMC13225991; doi:10.3389/fpubh.2026.1841849)
Supplement: Supplementary file 1 [file Data_Sheet_1.PDF]

Preparing future dentists for artificial intelligence: a cross-sectional study of perceptions and educational needs in China

Section I. Demographic information

1. Your age: ________
2. Your gender:
- Male
- Female
3. Your training stage:
- Undergraduate
- Master's student
- Doctoral student
4. Proficiency in using personal computers or smartphones:
- Very unskilled
- Somewhat unskilled
- Average
- Somewhat skilled
- Very skilled

Section II. Participants' awareness of AI applications in dentistry

1. How familiar are you with artificial intelligence (AI)?
- Very familiar
- Fairly familiar
- Neutral
- Unfamiliar
- Completely unfamiliar

2. How familiar are you with AI applications in dentistry?

- Very familiar
- Fairly familiar
- Neutral
- Unfamiliar
- Completely unfamiliar

3. Which developmental stage do you think AI currently occupies in dentistry?

- Conceptual stage, with minimal applications
- Preliminary application stage, mainly in diagnostic assistance or research
- Rapid development stage, with increasingly widespread applications
- Mature application stage, deeply integrated into clinical workflows
- Unclear

4. Have you ever used any artificial intelligence software, apps, or mini programs for dentistry?

- Proficient in commonly used artificial intelligence software
- Able to use some software features independently
- Unable to use the software independently; guidance is required
- Never used

5. How did you learn about the application of artificial intelligence in dentistry? (Multiple responses were allowed.)

- Professional academic journals or conferences
- Industry news or professional media
- Continuing education training or lectures
- Medical school curriculum
- Peer exchange
- Promotion by related product or software companies
- Social media or web searches
- Little or no understanding

6. Based on your understanding, in which specific areas of dentistry can AI be applied? (Multiple responses were allowed.)

- Oral imaging analysis
- Caries risk assessment and radiographic diagnosis
- Periodontal disease radiographic diagnosis and status assessment
- Early screening for oral cancer
- Orthodontic treatment plan design and simulation
- Implant surgery planning and surgical guide design
- Computer-aided design and manufacturing (CAD/CAM) of prostheses
- Radiological examination of jawbone lesions
- Intelligent management of patient medical records
- Virtual assistant/chatbot
- Clinical decision support system
- Teaching and training simulation
- I am not sure

7. In your opinion, which of the following stages of oral treatment are most suitable for the introduction of AI? (Multiple responses were allowed.)

- Diagnostic phase
- Treatment planning phase
- Treatment implementation phase
- Post-treatment outcome evaluation and follow-up tracking
- Patient communication and education
- Clinic management process
- I am not sure

8. What do you think is the potential of AI in enhancing the following aspects of dental care and treatment? Please select one option for each statement:

Question	Very low potential	Less potential	Average potential	Higher potential	Very high potential
Accuracy of diagnosis					
Speed of diagnosis					
Ability to detect oral					

diseases early					
Individualization and optimization of treatment					
Patient education and communication					
Clinicians' work efficiency					
Clinic experience					
Overall quality of medical services					
Long-term follow-up of oral health management					

Section III. Participants' attitudes toward and acceptance of AI applications in dentistry

1. In general, what is your stance on applying AI technology to the field of dental care?

- Very supportive
- Relatively supportive
- Neutral or indifferent
- A bit worried
- Inconclusive

2. Here are some potential concerns or opinions people may have about AI applications. How much do you personally agree with the following statements? Please select one option for each statement:

Question	Strongly disagree	Comparative disagreement	General	Basically agree	Totally agree

Technical reliability					
Data privacy and security					
Homogenization/dehumanization of healthcare					
Occupational substitution risk					
Interpretability/“black box” issues					
Access equity/digital divide					
Technological dependence					
Unclear regulatory and ethical norms					
Cost issues					

3. When AI makes a mistake in the course of treatment, who should be responsible?

- AI developers
- Doctors
- Medical organizations
- Developers, doctors, and medical organizations should share responsibility

4. Are you personally interested in learning or acquiring knowledge and skills related to the application of AI in the field of dentistry?

- Very interested
- Somewhat interested
- Generally interested
- Not really interested
- No interest at all
- Inconclusive

5. At which stage of study do you think a course on AI applications would be most helpful?

- It should be included in undergraduate dental education
- It should be included in master’s-level dental education

- It should be included in doctoral dental education
- It should be included in professional training for dentists
- Currently unknown

6. Are you willing to try applying AI tools or collaborating in the development of AI tools in future clinical practice or research?

- Very willing and looking forward to it
- More willing to try
- Neutral, depending on the specific situation
- Not really willing to try
- Very reluctant
- Inconclusive

7. When do you think AI will have a significant impact on the field of dentistry?

- Within 1 year
- Within 1–5 years
- Within 5–10 years
- More than 10 years
- Currently unknown

8. What is the most important aspect of dentistry that you would like to see AI address or improve?

- Improving diagnosis of certain diseases
- Optimizing complex surgical steps
- Reducing the paperwork burden
- Improving patient management efficiency
- Providing supplementary instruction
- Other
