# Supplementary material for: Preparing future dentists for artificial intelligence: a cross-sectional study of perceptions and educational needs in China
Source: Front Public Health. 2026 May 19;14:1841849. doi: 10.3389/fpubh.2026.1841849 (PMC13225991; doi:10.3389/fpubh.2026.1841849)
Supplement: Supplementary file 2 [file Data_Sheet_2.PDF]

Supplementary Table S1. Item-level classification and source mapping of questionnaire items

Question number	Item	Classification	Source	Notes
Section I, Q1	Age	Newly developed	This study	Standard demographic characteristic collected for participant description.
Section I, Q2	Gender	Newly developed	This study	Standard demographic characteristic.
Section I, Q3	Training stage	Newly developed	This study	Included to support stage-specific comparisons across undergraduate, master's, and doctoral students.
Section I, Q4	Proficiency in using personal computers or smartphones	Newly developed	This study	Background item used to describe participants' self-rated digital proficiency.
Section II, Q1	How familiar are you with artificial intelligence (AI)?	Substantially modified	[14]; informed by [15]	Based on prior constructs assessing general awareness or understanding of AI, but rewritten as a simple self-rated familiarity item.
Section II, Q2	How familiar are you with AI applications in dentistry?	Substantially modified	[14]; informed by [15]	Related to prior items on awareness of AI applications in medicine or dentistry, with wording adapted to the dental context.
Section II, Q3	Which developmental stage do you think AI currently occupies in dentistry?	Newly developed	This study	Dentistry-specific developmental-stage item without a clear direct counterpart in the cited source questionnaires.
Section II, Q4	Have you ever used any artificial intelligence software/apps/mini programs for dentistry?	Newly developed	This study	Use-experience item designed for the present study to reflect practical exposure to AI tools.
Section II, Q5	How did you learn about the application of artificial intelligence in dentistry?	Adapted	[10], [14]	Corresponds to prior questions on sources of AI information.

Section II, Q6	Based on your understanding, in which specific areas of dentistry can AI be applied?	Adapted	[10], [14]	Based on prior items regarding application fields or areas of benefit, expanded here into more detailed dental domains.
Section II, Q7	In your opinion, which of the following stages of oral treatment are most suitable for the introduction of AI?	Newly developed	This study	Newly designed item assessing perceived suitability of AI across stages of clinical workflow.
Section II, Q8	What do you think is the potential of AI in enhancing the following aspects of dental care and treatment?	Substantially modified	[14]; informed by [15]	Developed from prior attitude and usefulness constructs, but expanded into a dentistry-specific matrix covering multiple aspects of care.
Section III, Q1	In general, what is your stance on applying AI technology to the field of dental care?	Adapted	[10], [14]	General attitude item corresponding to previously published measures of AI acceptance.
Section III, Q2	Here are some potential concerns or opinions people may have about AI applications. How much do you personally agree with the following statements?	Adapted	[14]; informed by [15]	Based on previously published concern-related items, with refinement of specific concern domains for the present study.
Section III, Q3	When an AI makes a mistake in the course of treatment, who should be responsible?	Adapted	[10]	Closely aligned with prior items on responsibility attribution in the event of AI-related diagnostic or treatment errors.
Section III, Q4	Are you personally interested in learning or acquiring knowledge and skills related to the application of AI in the field of dentistry?	Adapted	[10], [14]	Reflects prior items addressing interest in AI-related education or training.
Section III, Q5	At which stage of study do you think a course on AI applications would be most helpful?	Substantially modified	[10], [14]	Derived from prior curriculum-related questions, but restructured to examine preferred educational stage.
Section III, Q6	Willingness to try to apply or collaborate on the development of AI tools in future clinical practice or research	Adapted	[10], [14]	Corresponds to prior questions on future willingness to use or engage with AI in professional settings.

Section III, Q7	When do you think AI will have a significant impact on the field of dentistry?	Adapted	[10]	Based on prior future-timeline questions, but reframed to assess anticipated impact rather than commercialization alone.
Section III, Q8	What is the most important aspect of dentistry that you would like to see AI address or improve?	Newly developed	This study	Priority-setting item designed specifically for the aims of the present study.

Notes:
Adapted = item retained the main construct and general wording logic of previously published questionnaires.
Substantially modified = item was informed by previously published questionnaire constructs but was materially rewritten for the present study.
Newly developed = item was developed specifically for the present study.
Reference [15] was used primarily to inform conceptual domain selection rather than as a direct item-level questionnaire source.
